# Fexofenadine Suppresses Delayed-Type Hypersensitivity in the Murine Model of Palladium Allergy

**DOI:** 10.3390/ijms18071357

**Published:** 2017-06-25

**Authors:** Ryota Matsubara, Kenichi Kumagai, Hiroaki Shigematsu, Kazutaka Kitaura, Yasunari Nakasone, Satsuki Suzuki, Yoshiki Hamada, Ryuji Suzuki

**Affiliations:** 1 Department of Oral and Maxillofacial Surgery, School of Dental Medicine, Tsurumi University, 2-3-1 Tsurumi, Tsurumi-ku, Yokohama 230-8501, Japan; matsubara-ryota@tsurumi-u.ac.jp (R.M.); shigematsu-h@tsurumi-u.ac.jp (H.S.); nakasone-yasunari@tsurumi-u.ac.jp (Y.N.); hamada-y@tsurumi-u.ac.jp (Y.H.); 2 Department of Rheumatology and Clinical Immunology, Clinical Research Center for Rheumatology and Allergy, Sagamihara National Hospital, National Hospital Organization, 18-1 Sakuradai, Minami-ku, Sagamihara 252-0392, Japan; k-kitaura@sagamihara-hosp.gr.jp; 3 Section of Biological Science, Research Center for Odontology, The Nippon Dental University School of Life Dentistry at Tokyo, 1-9-20 Fujimi, Chiyoda-ku, Tokyo 102-8159, Japan; satsukis@tky.ndu.ac.jp

**Keywords:** metal allergy, palladium, anti histamine, fexofenadine hydrochloride, corticosteroid

## Abstract

Palladium is frequently used in dental materials, and sometimes causes metal allergy. It has been suggested that the immune response by palladium-specific T cells may be responsible for the pathogenesis of delayed-type hypersensitivity in study of palladium allergic model mice. In the clinical setting, glucocorticoids and antihistamine drugs are commonly used for treatment of contact dermatitis. However, the precise mechanism of immune suppression in palladium allergy remains unknown. We investigated inhibition of the immune response in palladium allergic mice by administration of prednisolone as a glucocorticoid and fexofenadine hydrochloride as an antihistamine. Compared with glucocorticoids, fexofenadine hydrochloride significantly suppressed the number of T cells by interfering with the development of antigen-presenting cells from the sensitization phase. Our results suggest that antihistamine has a beneficial effect on the treatment of palladium allergy compared to glucocorticoids.

## 1. Introduction

Metal allergy is an inflammatory disease categorized as a delayed-type hypersensitivity (DTH) reaction, and is thought to be caused by the release of ions that function as haptens from metal materials [1]. Adverse skin reactions to metal ions, such as intractable dermatitis, pustulosis palmaris et plantaris, and incompatibility reactions to metal-containing biomaterials are serious problems [2,3,4]. It has been suggested that metal allergy is associated with the infiltration of lymphocytes into sites of allergic inflammation [5,6,7,8]. Among metals in biomaterials, palladium (Pd), which is frequently used in industry, jewelry, surgical instruments, dental implants [9], and dental materials (it is a common constituent of dental restorative alloys for crowns and bridges) [10,11], causes metal allergy [12]. The incidence of patients sensitized to Pd has increased in recent years [13,14,15].

Metal-induced DTH is driven by T cell sensitization to metal ions. T cells are largely responsible for the development of metal allergy in mice and humans [16,17]. We have previously generated a novel murine model of Pd allergy [6] and found an accumulation of Pd-specific T cells that exert cytotoxic effects and secrete inflammatory mediators to produce skin reactions at 7 days after the final challenge. It has been suggested that antihistamine drugs are effective for treatment of Pd-induced allergic contact dermatitis (ACD) mice within 24 h after the challenge [18]. However, the precise mechanism of inhibition for effective treatment of Pd allergy is unclear during the long period after the challenge.

Glucocorticoids and antihistamine drugs are the first selection for symptomatic treatment of contact dermatitis [19]. Although glucocorticoids are effective for most inflammatory skin disorders, their use is limited by local and systemic side effects [20]. Therefore, clinicians have to balance the benefits of treatment with glucocorticoids or antihistamines against the potential long-term detrimental effects on the DTH.

In the present study, we examined inhibition of the immune response in the Pd-induced murine model by administration of prednisolone as a glucocorticoid and fexofenadine hydrochloride as an antihistamine.

## 2. Results

### 2.1. Footpad Swelling in Pd-Induced Allergic Mice Administered with Fexofenadine or Prednisolone

**Figure 1:**
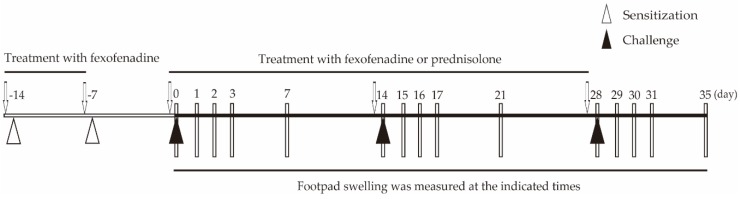
Schedule of the sensitization and elicitation of palladium (Pd)-induced allergic mice and oral administration of fexofenadine or prednisolone. The start of the first challenge was defined as day 0. Sensitization using palladium was performed every week throughout the experimental period from day –14 to day 0. The challenge for elicitation using Pd was performed every 2 weeks throughout the experimental period from day 0 to day 35. Arrows show when fexofenadine hydrochloride and prednisolone were orally administered at 10 mg/kg at 1 h before sensitization or challenge. Between challenges, the bar indicates the measurement day of left and right footpads. Footpad swelling was measured at 28, 29, 30, 31, and 35 days. All mice were sacrificed at day 35, and the footpads were taken as samples.

**Figure 2:**
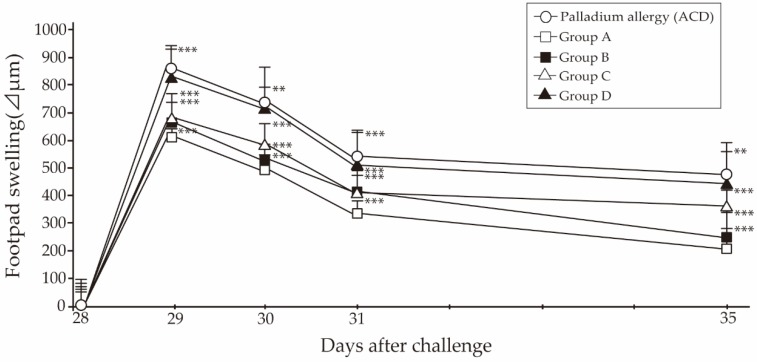
Effect of fexofenadine hydrochloride and prednisolone on footpad swelling with Pd induced allergy. Fexofenadine and prednisolone were orally administered to Pd-induced ACD mice at 1 h before sensitization or challenge. Furthermore, Pd-induced ACD mice received fexofenadine hydrochloride or prednisolone, and the mice were divided into four groups as shown in Table 2. ○, Pd-induced ACD; □, Group A; ■, Group B; △, Group C; ▲, Group D. Bars and error bars indicate the mean + standard deviation (SD). ** *p* < 0.01 is considered as very significant, and *** *p* < 0.001 is considered as extremely significant.

All experimental protocols were determined as described in the Materials and Methods (Figure 1, Table 1 and Table 2). To address how inflamed skin was inhibited by fexofenadine or prednisolone, we employed Pd-induced allergic contact dermatitis (ACD) mice (Figure 2). To determine the dose of fexofenadine and prednisolone, we have converted the approximate dose of human dosage into our Pd-induced ACD mice. In the preliminary experiment, the dose of fexofenadine (5 mg/kg) showed suppression equal to that shown by the dose of fexofenadine (50 mg/kg) in Pd-induced ACD mice (Figure S1A). Otherwise, the dose of prednisolone (5 mg/kg) showed the suppression of footpad swelling, but the dose of prednisolone (10 mg/kg) was more effective for suppression after each challenge (Figure S1B). Pd-induced ACD mice treated with fexofenadine (5 mg/kg, and 10 mg/kg) and prednisolone (5 mg/kg, and 10 mg/kg) did not show weight loss and poor fur condition, however, the Pd-induced ACD mice treated with prednisolone (30 mg/kg, and 50 mg/kg) showed weight loss, and poor fur condition (Figure S2). Thus, we determined the dose of fexofenadine and prednisolone as 10 mg/kg.

**Figure 3:**
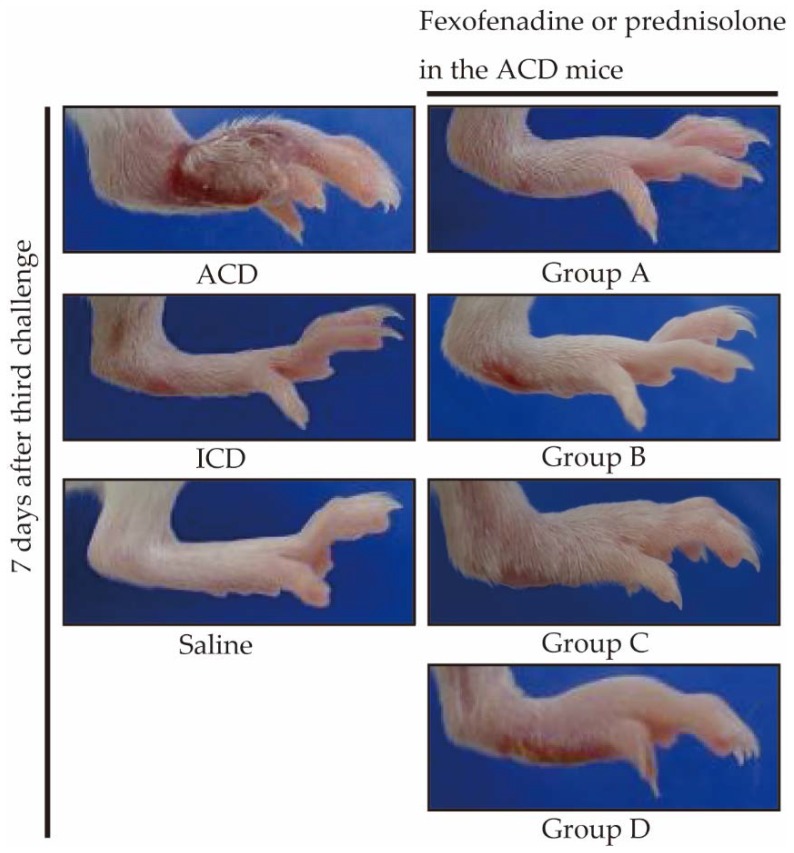
Macroscopic findings in Pd-induced ACD mice administered fexofenadine or prednisolone. Photographs show footpad swelling of a representative mouse at 7 days after the last challenge of Pd. In comparison with the footpad of Pd allergy mice, in terms of the redness and swelling, the feet of the groups A, B, C, and D showed decreases; in particular groups A and B showed dominant decreases.

In all groups, the peak of footpad swelling was observed at 24 h after challenge. Next, we examined whether administration of fexofenadine or prednisolone affected the footpad swelling. Administration of fexofenadine and prednisolone at 10 mg/kg/d significantly suppressed the increase in footpad swelling compared with untreated ACD mice (Figure 2). The administration of fexofenadine led to significant inhibition of the increase in footpad swelling compared with prednisolone. Regarding the administration of fexofenadine, the fexofenadine administration before challenge only (Group B) reduced the responses to a lesser degree than the fexofenadine administration before both sensitization and challenge (Group A). With respect to the administration of prednisolone, the prednisolone administration before challenge only (Group D) reduced the responses to a lesser degree than the fexofenadine administration before sensitization (Group C). As shown in a representative photograph, clear swelling and redness of the footpad were observed at 7 days after the last challenge, which were suppressed by fexofenadine administration (Figure 3).

### 2.2. Histological and Immunohistochemical Analyses of F4/80 and T cell Markers in Footpads of Pd-Induced Allergic Mice Administered with Fexofenadine or Prednisolone

**Figure 4:**
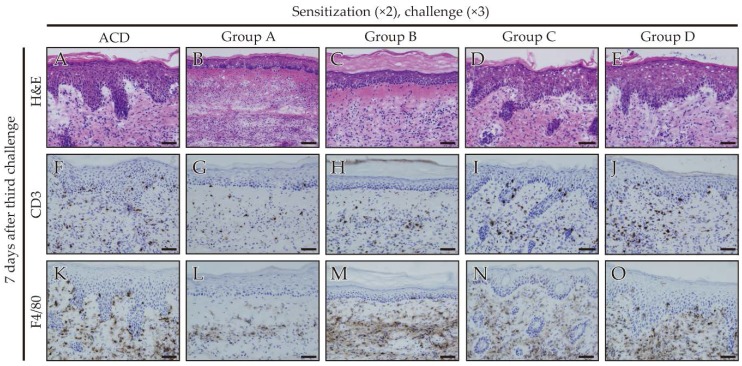
Histopathology and immunohistochemical analyses of accumulated T cells and antigen-presenting cells (APCs) in Pd-induced ACD mice treated with fexofenadine or prednisolone. Histopathology and immunohistochemical analyses of monoclonal antibody (mAb) that binds to a surface molecule on mature macrophages and dendritic cells (F4/80-positive cells) and cluster of differentiation (CD) 3-positive T cells in footpad tissues. Frozen footpad tissue sections were stained with hematoxylin and eosin (H&E) (**A**–**E**) and anti-CD3 (**F**–**J**) and anti-F4/80 (**K**–**O**) antibodies at 7 days after the last challenge. Scale bar = 100 µm.

**Figure 5:**
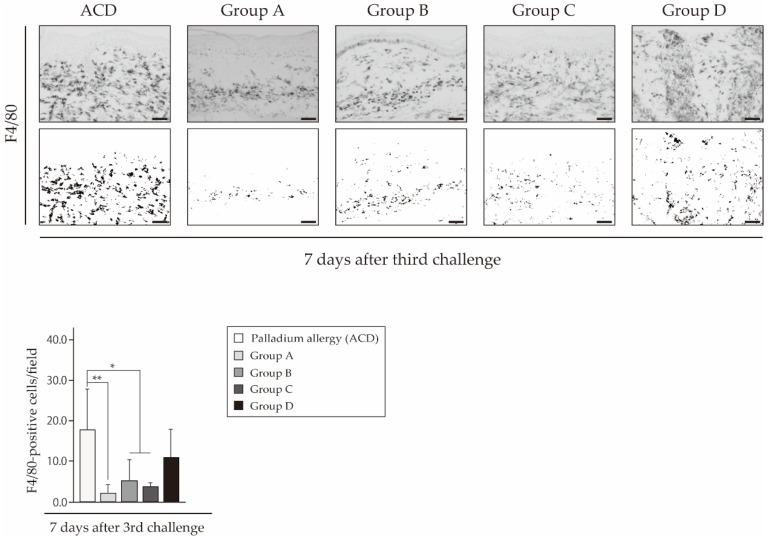
Imaging calculation of F4/80-positive cells. Measurements were made in six different regions of each sample, and mean values were used for statistical analysis. The figure show the representative photos of each group. The number of F4/80-positive cells at 7 days after the last challenge per target area was counted using ImageJ software with Java-based color deconvolution (v. 1.41). Bars and error bars indicate the mean + standard deviation (SD). * *p* < 0.05 is considered as significant, and ** *p* < 0.01 is considered as very significant. Scale bar = 100 µm.

To verify whether antigen-presenting cells (APCs) and T cells infiltrated into the site of inflamed skin, we analyzed the footpad skin of each mouse by immunohistochemistry. Hematoxylin and eosin (H&E) staining showed epithelial acanthosis, and epidermal spongiosis and liquefaction degeneration of the epithelial basal layer infiltrated with a dense mononuclear cells in the epithelial basal layer and upper dermis of ACD mice (Figure 4A,D,E). In particular, epidermal keratinocytes were separated partially (Figure 4A). The inflammatory reaction in the footpads was diminished in mice that received only fexofenadine (Figure 4B,C). Immunohistochemical staining showed that cluster of differentiation (CD) 3-positive T cells and F4/80-positive cells predominantly existed in the epithelial basal layer and upper dermis of ACD mice (Figure 4F,K). CD3-positive T cells were mainly present in the epithelial basal layer and upper dermis of mice that received prednisolone at a higher degree than in mice that received fexofenadine (Figure 4F–J). F4/80-positive cells had diminished in the epithelial basal layer of mice administered with fexofenadine compared with prednisolone (Figure 4K–O and Figure 5). Furthermore, administration of fexofenadine from sensitization significantly suppressed F4/80-positive cells in the epithelial basal layer (Figure 4L and Figure 5). In contrast, prednisolone did not suppress the infiltrating F4/80-positive cells (Figure 4O and Figure 5).

### 2.3. Expression Levels of T Cell Markers, Related Cytokines, and APC-Derived Signals Induced by Fexofenadine and Prednisolone

**Figure 6:**
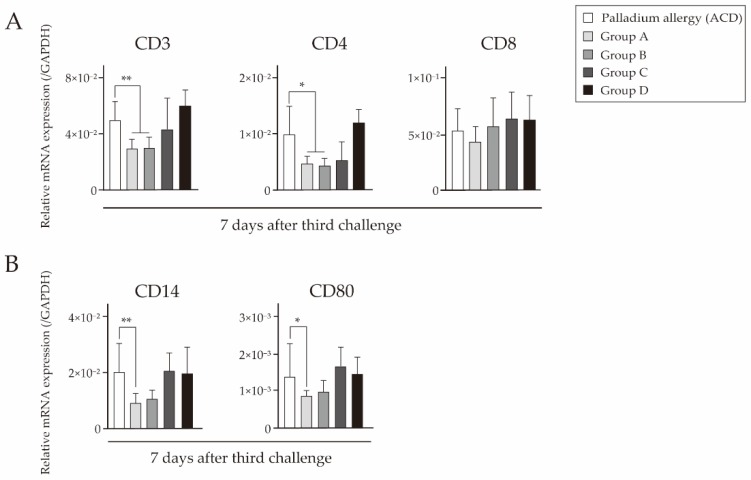
Effects of fexofenadine hydrochloride on mRNA expression levels indicating T cell phenotypes and T cell-related markers in Pd-induced ACD mice. Messenger RNA (mRNA) expression levels of CD3, CD4, CD8 (**A**), CD14, and CD80 (**B**) in footpads were assessed at 7 days after challenge. Glyceraldehyde-3-phosphate dehydrogenase (GAPDH) gene expression was used as an internal control. Bars and error bars indicate the mean + standard deviation (SD). Statistical significance was tested by the unpaired Mann Whitney test. * *p* < 0.05 is considered as significant and ** *p* < 0.01 is considered as very significant.

**Figure 7:**
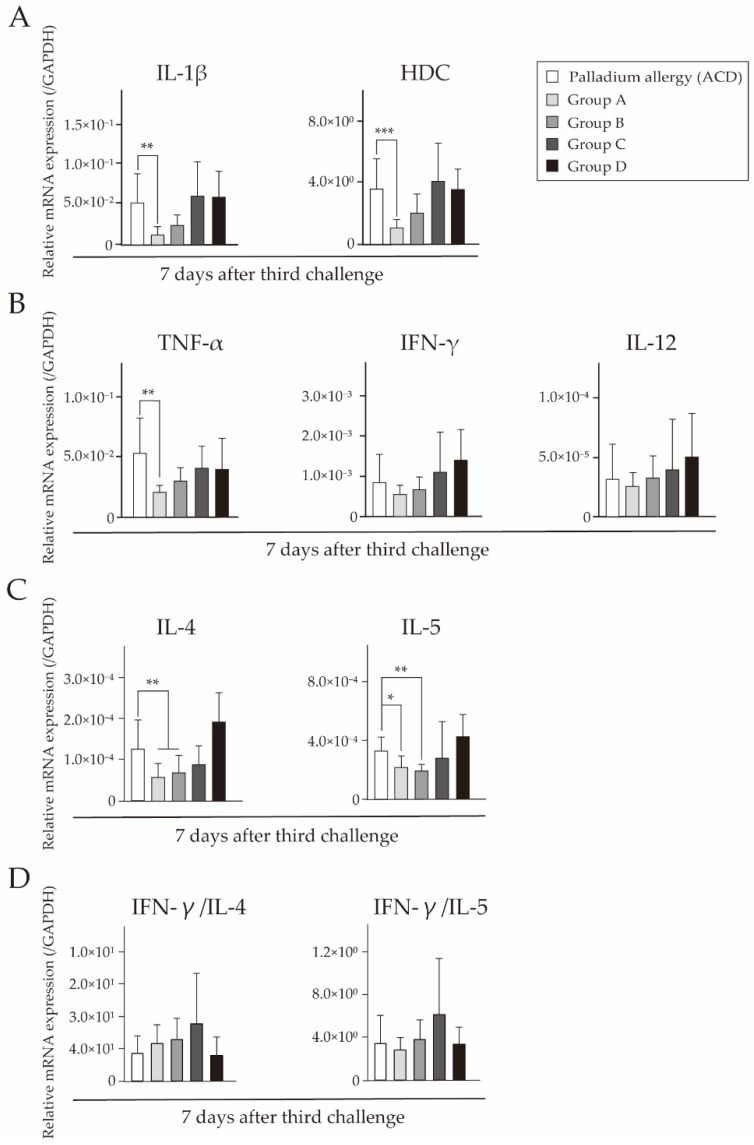
Effects of fexofenadine hydrochloride on the mRNA expression levels of T cell-related cytokines and chemokines in Pd-induced ACD mice. mRNA expression of (**A**) proinflammatory cytokine interleukin (IL) -1β, Histidine decarboxylase (HDC), (**B**) T helper type (Th) 1 cytokines (tumor necrosis factor (TNF) -α, Interferon (IFN) -γ, and IL-12), (**C**) T helper type (Th) 2 cytokines (IL-4 and IL-5), and (**D**) Th1/Th2 cytokine imbalance (IFN-γ/IL-4 and IFN-γ/IL-5) are shown. GAPDH gene expression was used as an internal control. Bars and error bars indicate the mean + standard deviation (SD). Statistical significance was tested by the unpaired Mann Whitney test. * *p*<0.05 is considered as significant, ** *p* < 0.01 is considered as very significant, and *** *p* < 0.001 is considered as extremely significant. TNF: tumor necrosis factor; HDC: histidine decarboxylase; IFN: interferon; IL: interleukin.

We investigated the expression levels of T cell markers, related cytokines, and APC-derived signals by quantitative polymerase chain reaction (qPCR). Cytokine expression at 7 days after challenge was measured in footpads of Pd-challenged mice. In mice that received fexofenadine, CD4 levels were significantly lower than in ACD mice. However, CD8 levels in skin were not significantly different (Figure 6A). Notably, mice that received fexofenadine before both sensitization and challenge had suppressed messenger RNA (mRNA) expression levels of APC-derived signals (Figure 6B). Prednisolone administration before challenge only did not affect T cell suppression. We also examined the expression levels of proinflammatory cytokine interleukin (IL)-1β, histidine decarboxylase (HDC), T helper type (Th) 1 cytokines (tumor necrosis factor (TNF)-α, interferon (IFN)-γ, and IL-12), Th2 cytokines (IL-4 and IL-5), and Th1/Th2 cytokine imbalance (IFN-γ/IL-4 and IFN-γ/IL-5). Mice that received fexofenadine before both sensitization and challenge had suppressed mRNA expression levels of IL-1β, HDC, and TNF-α (Figure 7A,B). Furthermore, mice that received only fexofenadine had suppressed mRNA expression levels of IL-4 and IL-5 (Figure 7C). However, there was no significant differences in the Th1/Th2 cytokine imbalance (Figure 7D). In contrast, mice that received prednisolone did not have suppressed mRNA expression levels of these T cell and APC markers.

## 3. Discussion

In this study, we demonstrated that fexofenadine inhibited immune responses of Pd allergy compared with glucocorticoids. Furthermore, the administration of fexofenadine hydrochloride during the sensitization phase significantly suppressed the number of T cells by interfering with the development of APCs in the elicitation phase.

It has been suggested that antihistamine drugs interfere with T cell-related inflammatory molecules at various points of the DTH immune cascade [21]. In our previous study, administration of antihistamine suppressed footpad swelling in the Pd allergy mouse model [18]. However, the precise mechanism of immunosuppression in Pd allergy remained unknown. In the present study, we investigated the suppressive effect of antihistamine in terms of the timing of administration compared with prednisolone, and found new aspects of antihistamine in metal allergy.

Metal allergy has two phases in the cutaneous hypersensitivity response: sensitization and elicitation. During the sensitization phase, cutaneous APCs take up and process antigens, and then migrate to regional lymph nodes where they activate T cells with consequent production of memory T cells, which localize in the dermis. In the elicitation phase, subsequent exposure to the sensitization chemical leads to antigen presentation to memory T cells in the dermis [22,23]. ACD is based on DTH reactions that involve antigen presentation by APCs and the T cell response [24]. Sensitization of metal-reactive T cells requires disruption of the barrier function and interaction of metal ions with major histocompatibility complex/peptide complexes presented by APCs to naive T cells [25]. Activation of APCs is essential for the establishment of sensitization with haptens [26]. Fexofenadine has been reported to suppress APC functions concerning skin immunity [27], whereas prednisolone did not. Thus, our results suggest that fexofenadine may act on APCs in DTH.

DTH led to spongiosis in the inflamed footpad skin and epithelial hyperplasia. Moreover, T cells are recruited to allergic sites in ACD lesions. In the present study, the administration of fexofenadine suppressed the development of CD3-positive T cells and F4/80-positive cells in the Pd-induced ACD mice (Figure 4 and Figure 5). In addition, apparent spongiosis changes, edema, and epithelial hyperplasia almost remained unaffected. The activated T cells cause edema and epidermal spongiosis [26]. These findings indicate that CD3-positive T cells were inhibited by fexofenadine in response to Pd in the skin of Pd-induced ACD mice. Local redness and swelling of footpads were followed by an increase in T cell numbers [28], which were also macroscopically found after fexofenadine administration. Therefore, fexofenadine was capable of inhibiting Pd allergy just prior to sensitization or elicitation (Figure 4G,H). F4/80 is a monoclonal antibody (mAb) that binds to a surface molecule on mature macrophages and dendritic cells. It is used to explore the role of epidermal and dermal cells as APCs during the induction of DTH in mice [29]. This study indicated that F4/80-positive cells were inhibited by fexofenadine administration before sensitization in response to Pd in the skin of Pd-induced ACD mice (Figure 4L). Fexofenadine administration before sensitization significantly suppressed the development of both CD3-positive T cells and F4/80-positive cells. A previous study has reported that olopatadine administration before sensitization reduces the DTH reaction to a lesser degree than olopatadine administration before challenge only [30].

We further investigated the cytokine expression profiles in the footpads of Pd-induced ACD mice. A previous study has suggested that Th1-type cytokines or Th1 and Th2 cytokines are preferentially produced in response to Pd [6,12]. It has been suggested that fexofenadine suppresses the infiltration of lymphocytes and Th2 cytokine production [31]. Fexofenadine treatment has prevented the development of allergy in challenge even in sensitized mice [31]. In this study, fexofenadine reduced the expression levels of CD3, CD4, IL-4, and IL-5. Thus, our results were consistent with previous studies. The hypersensitivity allergic inflammatory response involves histamine and Th2 cells. Histamine is synthesized by a catalytic enzyme called HDC. Mast cells and basophils are the most well-described cellular sources of histamine, but dendritic cells and T cells can also express HDC upon stimulation [32]. During the sensitization phase of DTH, cutaneous APCs migrate into skin-draining lymph nodes. It is known that the development of DTH is based on APC maturation and presentation of antigens to naive T cells in lymph nodes [33]. The maturation and migration of APCs are mainly regulated by TNF-α on mast cells [34]. Furthermore, mast cells and mast cell-associated TNF-α are supposed to be important contributors to the migration of hapten-bearing APCs from the initial stages of the sensitization phase of DTH [35]. Histamine not only alters APC and T cell functions, but also inhibits CD8 T cell proliferation [31], thereby deviating the immunoregulatory effects of the immune response to a Th2 response. Histamine enhances the secretion of Th2 cytokines, such as IL-4 and IL-5, and inhibits the production of Th1 cytokines such as IFN-γ and IL-12 [36]. Th2 cytokines were suppressed because fexofenadine inhibits histamine. Hence, treatment with fexofenadine prevented the increases in IL-4 and IL-5 levels, but IFN-γ levels were hardly affected (Figure 7B,C). The Th1/Th2 cytokine imbalance with a predominance of Th2 cytokines is known to be crucial for the pathogenesis of hypersensitivity allergic diseases [21].Fexofenadine controls host immune responses by suppressing Th2 responses, and also affects the imbalance of Th1/Th2 cytokines (Figure 7D). Therefore, modulation of the Th1/Th2 cytokine imbalance is a promising strategy for the treatment of hypersensitivity allergic diseases.

Current symptomatic treatment for metal allergy employs antihistamines or general immune suppressants such as corticosteroids [37]. Corticosteroids have various inhibitory actions in the immune system as well as harmful effects. Both immediate hypersensitivity and DTH reactions are rare in patients treated with systemic corticosteroids. However, delayed-type reactions to systemically administered steroids may present as a generalized dermatitis [38]. Although steroids are used as first-line therapy for allergic diseases, they can induce ACD in sensitized patients [39]. The therapeutic use of corticosteroids requires a careful balance between helping the patient by reducing inflammatory manifestations of the disease and causing harm from the toxic side-effects [40]. For this reason, allergic disease is often treated in combination with other drugs to keep the doses and toxic effects to a minimum. Fexofenadine hydrochloride effectively inhibits histamine-induced cutaneous wheals more than prednisolone [41,42]. Histamine is the major mediator of acute inflammatory and immediate hypersensitivity responses, and has been suggested to affect chronic inflammation [43]. Fexofenadine suppresses not only histamine, but also APC functions concerning skin immunity [27]. Histamine plays a modulatory role in the cortical arousal system mainly through the H1 receptor (H1R). The fexofenadine inhibited H1R occupancy in the human brain than the other antihistamine, therefore, fexofenadine hydrochloride has high safety compared with other antihistamines [44,45]. For allergic responses, the best treatment is avoidance of the allergen, which is not always possible. Although the unwanted immune responses that occur in allergy present somewhat difficult problems, the therapeutic goal in all cases is to inhibit the harmful immune response and thus avoid damage to tissues or disruption of their function [46]. We elucidated immune suppression of fexofenadine in Pd allergy, and demonstrated the inhibitory activities of APCs and infiltrating T cells in Pd allergy. Our data may indicate the clinical utility of fexofenadine as a candidate therapeutic drug for Pd allergy.

## 4. Materials and Methods

### 4.1. Ethics Statement

This study was performed in strict accordance with the recommendations in the Guidelines for Care and Use of Laboratory Animals of Tsurumi University and the Clinical Research Center of Sagamihara National Hospital, Japan. All animal experiments were performed according to the relevant ethical requirements with approval from the committees for animal experiments at Tsurumi University (approval number 27A061, issued on 9 June 2015) and the Clinical Research Center for Rheumatology and Allergy, Sagamihara National Hospital (approval number H22-2010-1, issued on 31 March 2010). All surgeries were performed under three types of mixed anesthetic agents, and all efforts were made to minimize suffering.

### 4.2. Animals

BALB/cAJcl mice (4-week-old females, each experimental group included six mice, average weight = 18.3 g) were purchased from CLEA Japan (Tokyo, Japan). During the study period, all mice remained in good health, and they were assigned randomly to various groups. Animals were acclimated for at least 7 days before experimental use. Mice at 11–12 weeks of age were used for experiments, and their weights ranged from 22.7 to 25.0 g (average 24.1 g). All mice were kept in plastic cages with a lid made of stainless steel wire at our conventional animal facility that maintained the temperature at 19–23°C and humidity at 30–70% with a 12-h day/night cycle. Food and water were available ad libitum.

### 4.3. Reagents

PdCl_2_ (>99% pure) was purchased from Wako Pure Chemical Industries (Osaka, Japan). Lipopolysaccharide (LPS) from *Escherichia coli* (O55:B5) prepared by phenol–water extraction was purchased from Sigma (St Louis, MO, USA). Prednisolone (>99% pure) was purchased from Sigma-Aldrich Japan (Tokyo, Japan). Fexofenadine hydrochloride (>98% pure) was purchased from Tokyo Chemical Industry Co., Ltd. (Tokyo Japan). PdCl_2_, prednisolone, fexofenadine hydrochloride, and LPS were dissolved in sterile saline. 

### 4.4. Anesthetic Agents

Medetomidine hydrochloride was purchased from Nippon Zenyaku Kogyo Co., Ltd. (Fukushima, Japan). Midazolam was purchased from Sandoz (Tokyo, Japan). Butorphanol tartrate was purchased from Meiji Seika Pharma Co., Ltd. (Tokyo, Japan). These anesthetics were kept at room temperature (RT). Three types of mixed anesthetic agents were prepared with medetomidine hydrochloride at a dose of 0.3 mg/kg, midazolam at a dose of 4 mg/kg, and butorphanol tartrate at a dose of 5 mg/kg. The concentration ratio of the three types of mixed anesthetic agents was determined by a previous study [47]. A total of 0.75 mL medetomidine hydrochloride was mixed with 2 mL midazolam and 2.50 mL butorphanol tartrate, and adjusted to a volume of 19.75 mL with sterile saline. All agents were diluted in sterile saline and stored at 4 °C in the dark. The mixed anesthetic agents were administered to mice at a volume of 0.01 mL/g of body weight.

### 4.5. Experimental Protocol

Based on previous reports [6,48], we established the experimental protocols (Figure 1). Each experimental group of mice was separated into seven sets with each set consisting of six randomly chosen mice. All experiments were carried out in another room after transfer from the animal holding room.

Sensitization: A total of 125 µL of 10 mM PdCl_2_ and 10 μg/mL LPS in sterile saline was injected twice at an interval of 7 days via the intradermal (i.d.) route into the left and right groin of mice (250 µL each). At 7 days after the second sensitization, mice were challenged for the first time.

Challenge for elicitation: At day 7 after the second sensitization, non-sensitized mice (ICD mice) or sensitized mice (ACD mice) were challenged for elicitation with 25 µL of 10 mM PdCl_2_ without LPS in sterile saline into the left and right footpad by i.d. injection under anesthesia with three types of mixed anesthetic agents. Mice sensitized with Pd plus LPS and then challenged with sterile saline were used as the control (Table 1). After weighing the mice, the appropriate volumes of the three types of mixed anesthetic agents were administered by intraperitoneal injection into the lower left or right quadrant of each mouse under manual restraint.

### 4.6. Oral Administration of Fexofenadine Hydrochloride and Prednisolone

Pd allergy was induced in mice as described above. On the day of each challenge, each drug (fexofenadine hydrochloride, or prednisolone) was orally administered to each ACD mouse once daily at 1 h before challenge. In some experimental ACD mouse groups, the dose of fexofenadine hydrochloride was orally administered at 1 h before sensitization. The mice received 10 mg/kg fexofenadine hydrochloride or prednisolone via a gastric tube. The dose of fexofenadine hydrochloride or prednisolone were 0.01 mg/g of weight in 100 µL of sterile saline. The approximate dose indicated that we converted from murine weight for humans. Control and ICD mice received the same volume of sterile saline alone. ACD mice were divided into four groups: Group A, fexofenadine hydrochloride was orally administered at 1 h before sensitization and challenge; Group B, fexofenadine hydrochloride was orally administered at 1 h before challenge only; Group C, fexofenadine hydrochloride was orally administered at 1 h before sensitization and prednisolone was orally administered at 1 h before challenge; and Group D, prednisolone was orally administered at 1 h before challenge only (Table 2).

### 4.7. Measurement of Allergic Footpad Swelling

Footpad swelling was measured before challenge and at 24 h, 48 h, and 72 h, and 1 week after challenge using a Peacock dial thickness gauge (Ozaki MFG Co. Ltd., Tokyo, Japan). The difference in footpad thickness before and after challenge was recorded. All procedures were performed by the same operator.

### 4.8. Immunohistochemistry

Footpads were obtained from Pd-induced ACD mice, fexofenadine hydrochloride-treated mice, and prednisolone-treated mice for histology and immunohistochemical analyses. Tissue samples were fixed with 4% paraformaldehyde-lysine-periodate overnight at 4 °C. After washing with phosphate buffered saline (PBS), fixed tissues were soaked in 5% sucrose/PBS for 1 h, 15% sucrose/PBS for 3 h, and then 30% sucrose/PBS overnight at 4 °C. Tissue samples were embedded in Tissue Mount (Chiba Medical, Saitama, Japan) and snap-frozen in a mixture of acetone and dry ice. Frozen sections were cut into 6-µm-thick cryosections that were air dried on poly-l-lysine-coated glass slides. For histological analyses, the cryosections were stained with H&E. For immunohistochemical analyses, antigen retrieval was performed, and the cryosections were stained with anti-mouse F4/80 (1:1000; Cl-A3-1, Abcam, Cambridge, UK) and anti-CD3 (1:500; SP7, Abcam, Cambridge, UK) mAbs. The F4/80 monoclonal antibody has been used to detect mouse macrophages populations in a wide range of footpad tissue. Non-specific binding of mAbs was blocked by incubation of the sections in PBS containing 5% normal goat rabbit serum, 0.025% Triton X-100 (Wako Pure Chemicals, Osaka, Japan), and 5% bovine serum albumin (Sigma-Aldrich) for 30 min at RT. The sections were incubated with primary mAbs for 1 h at RT. After washing three times with PBS for 5 min each, intrinsic peroxidase was quenched using 3% H_2_O_2_ in methanol. After soaking the sections in distilled water, they were washed twice and then incubated with a secondary antibody (biotinylated goat anti-hamster immunoglobulin G (IgG) or biotinylated rabbit anti-rat IgG) for 1 h at RT. After washing three times, the sections were treated with Vectastain ABC Reagent (Vector Laboratories, Burlingame, CA, USA) for 30 min at RT, followed by 3,3-diaminobenzidine (DAB) staining (0.06% DAB and 0.03% H_2_O_2_ in 0.1 M Tris-HCl, pH 7.6; Wako Pure Chemicals, Osaka, Japan). The tissue sections were counterstained with hematoxylin to visualize nuclei.

Images of F4/80-positive cells in immunostained sections were obtained using a BX 51 microscope (Olympus Optical, Tokyo, Japan) at ×200 magnification and stored in TIF format (4080 × 3072 resolution, 95 dpi). Selected tissue regions were delineated and subtracted from the respective layer area. Images were then thresholded to highlight the stained areas, but not the respective isotype controls. The highlighted section was analyzed and presented as a fraction of the selected region. To obtain representative results, measurements were made in six different regions of each sample, and mean values were used for statistical analysis. The number of positively immunostained cells per target area was counted using ImageJ software with Java-based color deconvolution (v. 1.41, US National Institutes of Health).

### 4.9. RNA Extraction and cDNA Synthesis

Fresh footpads were obtained from mice and immediately soaked in RNAlater RNA Stabilization Reagent (Qiagen, Hilden, Germany). Total RNA from footpads and spleens was extracted using the RNeasy Lipid Tissue Mini Kit (Qiagen, Hilden, Germany) according to the manufacturer’s instructions. cDNA was synthesized from DNA-free RNA using the PrimeScript™ RT reagent Kit (Takara Bio, Tokyo, Japan) according to the manufacturer’s instructions.

### 4.10. qPCR

The expression levels of immune response-related genes, including T cell-related CD antigens, cytokines, and cytotoxic granules, were measured by qPCR using the Bio-Rad CFX96 system (Bio-Rad, Hercules, CA, USA). Specific primers for GAPDH, CD3, CD4, CD8, CD14, CD80, IL-1β, IFN-γ, TNF-α, IL-4, IL-5, IL-12, and HDC have been described previously [7,49,50]. Freshly isolated total RNA from the footpads of mice was converted to complementary DNA (cDNA). The PCR consisted of 5 µL SsoFast™ EvaGreen^®^ Supermix (Bio-Rad), 3.5 µL RNase/DNase-free water, 0.5 µL of 5 µM primer mix, and 1 µL cDNA in a final volume of 10 µL. Cycling conditions were as follows: 30 s at 95 °C followed by 45 cycles of 1 s at 95 °C and 5 s at 60 °C. At the end of each program, melting curve analysis was performed from 65 °C to 95 °C to confirm the homogeneity of PCR products. All assays were repeated three times, and mean values were used to calculate gene expression levels. Five 10-fold serial dilutions of each standard transcript were used to determine the absolute quantification, specification, and amplification efficiency of each primer set. Standard transcripts were generated by in vitro transcription of the corresponding PCR product in a plasmid. The nucleotide sequences were confirmed by DNA sequencing using the CEQ8000 Genetic Analysis System (Beckman Coulter, Fullerton, CA, USA). Their quality and concentration were validated using an Agilent DNA 7500 Kit in an Agilent 2100 Bioanalyzer (Agilent, Santa Clara, CA, USA). GAPDH gene expression was used as an internal control. The expression levels of each target gene were normalized to GAPDH expression.

### 4.11. Statistical Analysis

The statistical significance of differences between mean values of each experimental group was analyzed using the Mann Whitney test by GraphPad Prism 5 software for Windows (GraphPad Software, Inc., San Diego, CA, USA). A *p*-value of less than 0.05 was considered as significant, a *p*-value of less than 0.01 was considered as highly significant, and a *p*-value of less than 0.001 was considered as extremely significant.

## 5. Conclusions

Our results show that the interference with the development of antigen-presenting cells by fexofenadine has the beneficial effect on the treatment of palladium allergy compared to prednisolone.

## Figures and Tables

**Table 1:** Experimental groups of contact dermatitis to Pd-induced allergy.

BALB/cAJcl	Sensitization	Challenge for Elicitation
ACD	+	+
ICD	−	+
Saline	+	−

ACD: allergic contact dermatitis; ICD: irritant contact dermatitis.

**Table 2:** Experimental groups of suppression of contact dermatitis to Pd-induced allergy by oral administration of fexofenadine or prednisolone.

Group	Sensitization	Challenge for Elicitation
A	F *	F
B	−	F
C	F	P **
D	−	P

* Fexofenadine hydrochloride; ** Prednisolone.

## Supplementary Materials

The following are available online at www.mdpi.com/1422-0067/18/7/1357/s1.
