# Supplementary material for: Fexofenadine Suppresses Delayed-Type Hypersensitivity in the Murine Model of Palladium Allergy
Source: Int J Mol Sci. 2017 Jun 25;18(7):1357. doi: 10.3390/ijms18071357 (PMC5535850; doi:10.3390/ijms18071357)
Supplement: Supplementary file 1 [file ijms-18-01357-s001.pdf]

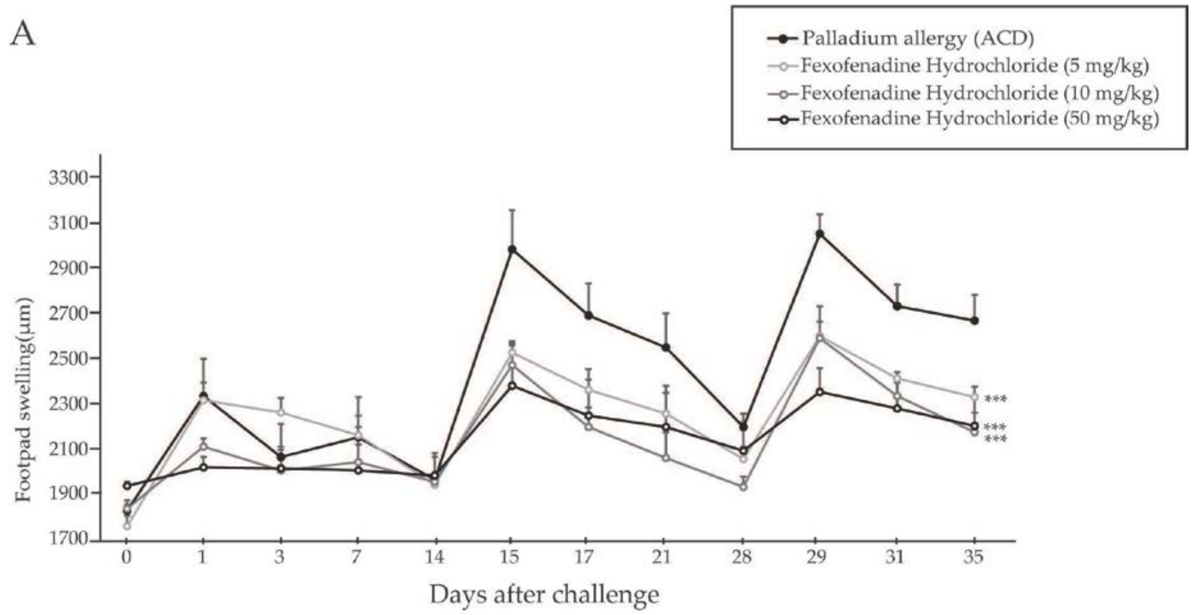

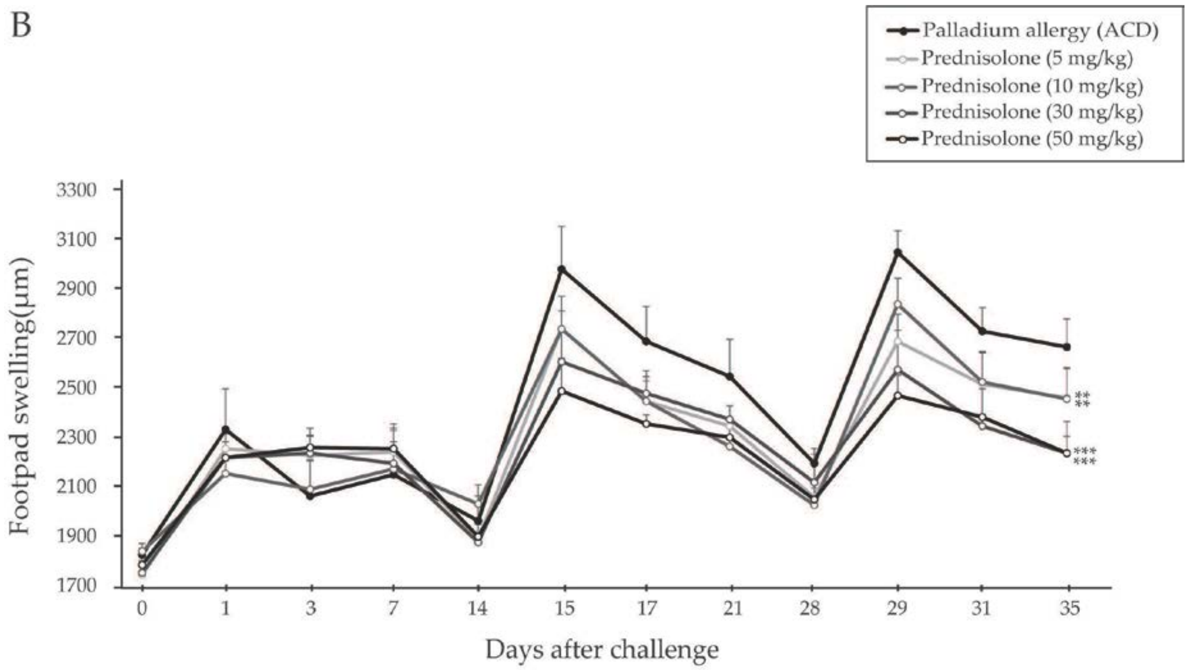

Figure S1. Footpad swelling at each dose of fexofenadine hydrochloride and prednisolone in the Pd-induced ACD mice. The dose of fexofenadine (5mg/kg) showed the suppression of footpad swelling, but the dose of fexofenadine (10mg/kg) had more effective for suppression in Pd-induced ACD mice (**A**). Otherwise, the dose of prednisolone (5mg/kg) was not enough to suppress Pd-induced ACD mice after the first and second challenges (**B**). Bars and error bars indicate the mean + standard deviation (SD). * $p < 0.05$ is considered as significant, ** $p < 0.01$ is considered as very significant, and *** $p < 0.001$ is considered as extremely significant.

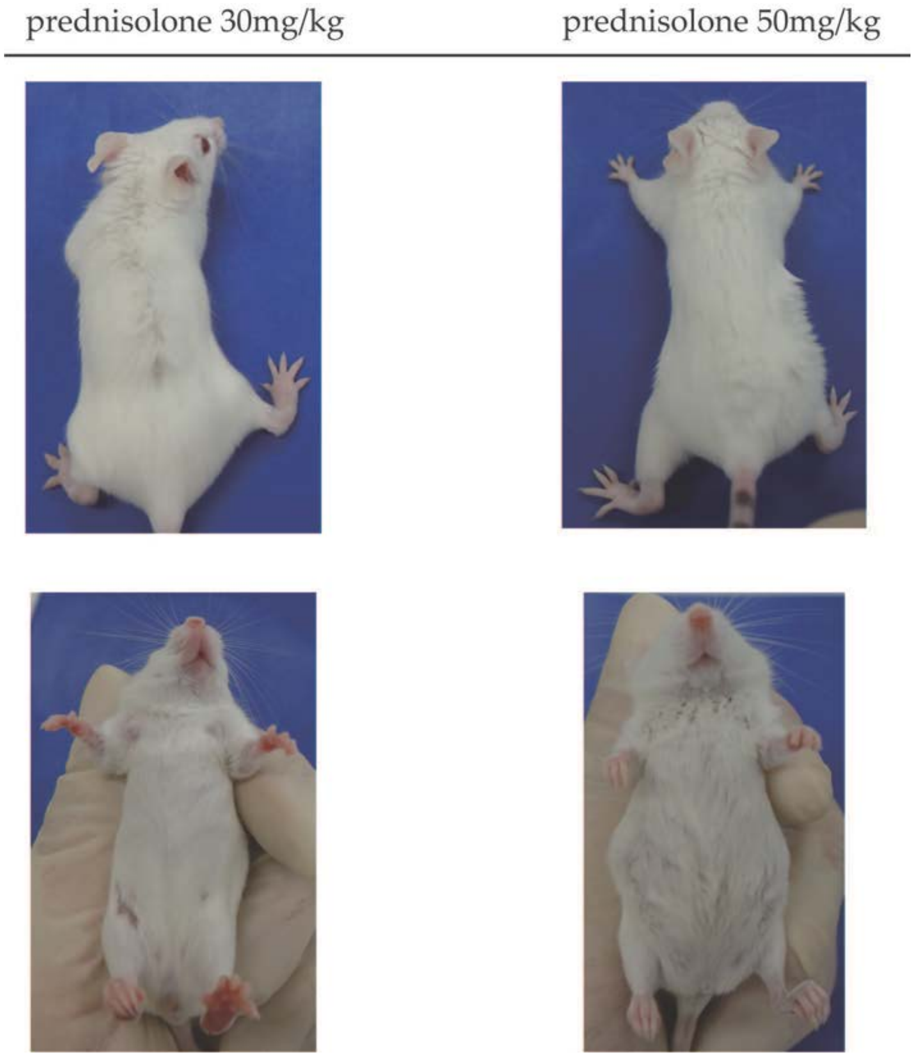

Figure 2. Macroscopic findings of Pd-induced allergic mice treated with high dose prednisolone. Pd-induced ACD mice treated with prednisolone at 30mg/kg and 50mg/kg show the bad fur condition.
